# *N*,*N*-Bis(2,6-difluoro­benz­yl)-1,3,4-thia­diazol-2-amine

**DOI:** 10.1107/S1600536809031675

**Published:** 2009-08-15

**Authors:** Hoong-Kun Fun, Wei-Ching Liew, B. Chandrakantha, Arun M. Isloor

**Affiliations:** a X-ray Crystallography Unit, School of Physics, Universiti Sains Malaysia, 11800 USM, Penang, Malaysia; b Syngene International Ltd, Biocon Park, Plot Nos. 2 & 3, Bommasandra 4th Phase, Jigani Link Rd, Bangalore 560 100, India; c Department of Chemistry, National Institute of Technology-Karnataka, Surathkal, Mangalore 575 025, India

## Abstract

In the title compound, C_16_H_11_F_4_N_3_S, the dihedral angles between the thia­diazole ring and the difluorobenzyl rings are 81.95 (7) and 81.96 (7)°, whereas the dihedral angle between the difluorobenzyl rings is 11.41 (7)°. In the crystal structure, C—H⋯N and C—H⋯F inter­actions link the mol­ecules into two-dimensional arrays parallel to the *bc* plane.

## Related literature

For the synthesis of pharmaceutically condensed heterocyclic thia­diazole derivatives as anti­microbials, see: Swamy *et al.* (2006 ▶). For the synthesis and anti-inflammatory, analgesic, ulcerogenic and lipid peroxidation activity of some new acetic acid derivatives, see: Amir & Shikha (2004 ▶). For new bis-amino­mercaptotriazoles and bis-triazolothia­diazo­les as possible anti­cancer agents, see: Holla *et al.* (2002 ▶). For the synthesis and biological evaluation of thia­diazole derivatives as a novel class of potential anti-tumor agents, see: Ibrahim (2009 ▶). For related structures, see: Wang *et al.* (2009*a* ▶,*b* ▶); Yin *et al.* (2008 ▶). For the stability of the temperature controller used in the data collection, see: Cosier & Glazer (1986 ▶).
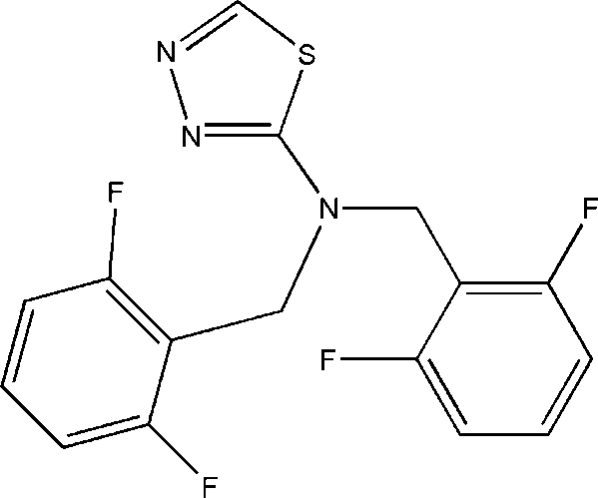


## Experimental

### 

#### Crystal data

C_16_H_11_F_4_N_3_S
*M* *_r_* = 353.34
Orthorhombic, 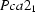

*a* = 32.6678 (6) Å
*b* = 5.8515 (1) Å
*c* = 7.8140 (2) Å
*V* = 1493.69 (5) Å^3^
*Z* = 4
Mo *K*α radiation
μ = 0.26 mm^−1^
*T* = 100 K
0.46 × 0.33 × 0.14 mm

#### Data collection

Bruker SMART APEXII CCD area-detector diffractometer
Absorption correction: multi-scan (**SADABS**; Bruker, 2005 ▶) *T*_min_ = 0.887, *T*_max_ = 0.965
17980 measured reflections
4796 independent reflections
4299 reflections with *I* > 2σ(*I*)
*R*_int_ = 0.027

#### Refinement

*R*[*F* ^2^ > 2σ(*F* ^2^)] = 0.033
*wR*(*F* ^2^) = 0.076
*S* = 1.02
4796 reflections
218 parameters
1 restraint
H-atom parameters constrained
Δρ_max_ = 0.23 e Å^−3^
Δρ_min_ = −0.26 e Å^−3^
Absolute structure: Flack (1983 ▶), 1935 Friedel pairs
Flack parameter: 0.20 (5)

### 

Data collection: *APEX2* (Bruker, 2005 ▶); cell refinement: *SAINT* (Bruker, 2005 ▶); data reduction: *SAINT*; program(s) used to solve structure: *SHELXTL* (Sheldrick, 2008 ▶); program(s) used to refine structure: *SHELXTL*; molecular graphics: *SHELXTL*; software used to prepare material for publication: *SHELXTL* and *PLATON* (Spek, 2009 ▶).

## Supplementary Material

Crystal structure: contains datablocks global, I. DOI: 10.1107/S1600536809031675/tk2527sup1.cif

Structure factors: contains datablocks I. DOI: 10.1107/S1600536809031675/tk2527Isup2.hkl

Additional supplementary materials:  crystallographic information; 3D view; checkCIF report

## Figures and Tables

**Table 1:** Hydrogen-bond geometry (Å, °)

*D*—H⋯*A*	*D*—H	H⋯*A*	*D*⋯*A*	*D*—H⋯*A*
C1—H1*A*⋯N1^i^	0.93	2.58	3.392 (2)	146
C3—H3*B*⋯F3^ii^	0.97	2.47	3.0226 (16)	116
C8—H8*A*⋯F4^iii^	0.93	2.54	3.144 (2)	123
C10—H10*B*⋯N2^iv^	0.97	2.56	3.4191 (17)	148

Symmetry codes: (i) 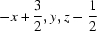; (ii) 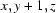; (iii) 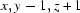; (iv) 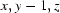.

**Fig. 1.**
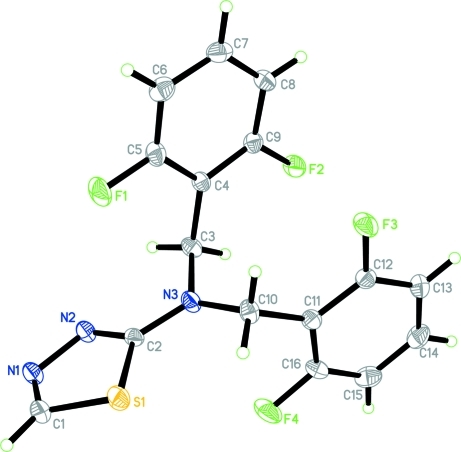
The molecular structure of (I), showing 50% probability displacement ellipsoids and the atom numbering scheme.

**Fig. 2.**
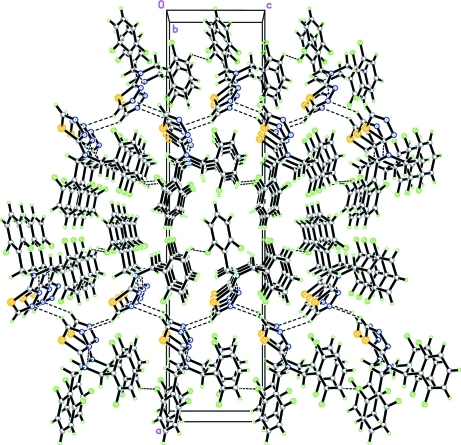
Crystal packing viewed along the b-axis. C-H···N and C-H···F interactions are shown as dashed lines.

## supplementary crystallographic information

### Comment

Heterocycles bearing 1,3,4-thiadiazole moieties represent an interesting class of compounds possessing a wide spectrum of biological activity (Swamy *et al.*, 2006) such as anti-inflammatory, anti-viral and anti-microbial properties (Amir & Shikha, 2004). Recently, 1,3,4-thiadiazoles have attracted particular attention due to their analgesic, ulcerogenic and lipidperoxidation activities (Holla *et al.*, 2002). In particular, the derivatives of variously substituted 1,3,4-thiadiazoles are also known to exhibit anti-depressant and anxiolitic agents (Ibrahim, 2009). Herein, the crystal structure of the title compound (I) is reported.

In (I), Fig. 1, the bond lengths and angles are comparable to related structures (Wang *et al.*, 2009*a* & *b*; Yin *et al.*, 2008). The dihedral angles between the thiadiazole ring (C1—C2/S1/N1—N2) and the difluorobenzyl rings [(C4—C9) and (C11—C16)] are 81.95 (7)° and 81.86 (7)°, respectively whereas the dihedral angle between the difluorobenzyl rings is 11.41 (7)°. These data indicate that all the three rings are twisted from each other.

The crystal packing (Fig. 2) is consolidated by C—H···N and C—H···F interactions (Table 1) that link molecules into two-dimensional arrays parallel to the *bc* plane.

### Experimental

Anhydrous potassium carbonate (5.4 g, 0.0395 mol) was added to a solution of 1,3,4-thiadiazole-2-amine (2 g, 0.0197 mol) in dry acetonitrile (25 ml). The reaction mixture was stirred for 15 min. 2,6-Difluorobenzyl bromide (8.18 g, 0.0395 mol) was added drop-wise to the mixture. After addition, the reaction mixture was stirred at room temperature for 18 h, filtered, and the filtrate was concentrated. The crude product was purified by column chromatography using 60–120 silica gel. The fraction eluted with 10% ethyl acetate in hexane was concentrated to produce (I) as a pale-yellow crystalline solid. Yield 5.0 g, 71.63%. *m.p.* 421–423 K.

### Refinement

C-bound H atoms were positioned geometrically [C—H = 0.93 and 0.97 Å] and refined using a riding model with *U*_iso_(H) = 1.2*U*_eq_(C).

### Crystal data

**Table:**

C_16_H_11_F_4_N_3_S	*F*(000) = 720
*M**_r_* = 353.34	*D*_x_ = 1.571 Mg m^−^^3^
Orthorhombic, *P**c**a*2_1_	Mo *K*α radiation, λ = 0.71073 Å
Hall symbol: P 2c -2ac	Cell parameters from 9387 reflections
*a* = 32.6678 (6) Å	θ = 2.9–33.7°
*b* = 5.8515 (1) Å	µ = 0.26 mm^−^^1^
*c* = 7.8140 (2) Å	*T* = 100 K
*V* = 1493.69 (5) Å^3^	Block, pale-yellow
*Z* = 4	0.46 × 0.33 × 0.14 mm

### Data collection

**Table:**

Bruker SMART APEXII CCD area-detector diffractometer	4796 independent reflections
Radiation source: fine-focus sealed tube	4299 reflections with *I* > 2σ(*I*)
graphite	*R*_int_ = 0.027
φ and ω scans	θ_max_ = 32.5°, θ_min_ = 2.5°
Absorption correction: multi-scan (*SADABS*; Bruker, 2005)	*h* = −48→46
*T*_min_ = 0.887, *T*_max_ = 0.965	*k* = −8→8
17980 measured reflections	*l* = −11→11

### Refinement

**Table:**

Refinement on *F*^2^	Secondary atom site location: difference Fourier map
Least-squares matrix: full	Hydrogen site location: inferred from neighbouring sites
*R*[*F*^2^ > 2σ(*F*^2^)] = 0.033	H-atom parameters constrained
*wR*(*F*^2^) = 0.076	*w* = 1/[σ^2^(*F*_o_^2^) + (0.0345*P*)^2^ + 0.2893*P*] where *P* = (*F*_o_^2^ + 2*F*_c_^2^)/3
*S* = 1.02	(Δ/σ)_max_ = 0.001
4796 reflections	Δρ_max_ = 0.23 e Å^−^^3^
218 parameters	Δρ_min_ = −0.26 e Å^−^^3^
1 restraint	Absolute structure: Flack (1983), 1935 Friedel pairs
Primary atom site location: structure-invariant direct methods	Flack parameter: 0.20 (5)

### Special details

**Table:**

Experimental. The crystal was placed in the cold stream of an Oxford Cyrosystems Cobra open-flow nitrogen cryostat (Cosier & Glazer, 1986) operating at 100.0 (1) K.
Geometry. All e.s.d.'s (except the e.s.d. in the dihedral angle between two l.s. planes) are estimated using the full covariance matrix. The cell e.s.d.'s are taken into account individually in the estimation of e.s.d.'s in distances, angles and torsion angles; correlations between e.s.d.'s in cell parameters are only used when they are defined by crystal symmetry. An approximate (isotropic) treatment of cell e.s.d.'s is used for estimating e.s.d.'s involving l.s. planes.
Refinement. Refinement of *F*^2^ against ALL reflections. The weighted *R*-factor *wR* and goodness of fit *S* are based on *F*^2^, conventional *R*-factors *R* are based on *F*, with *F* set to zero for negative *F*^2^. The threshold expression of *F*^2^ > σ(*F*^2^) is used only for calculating *R*-factors(gt) *etc*. and is not relevant to the choice of reflections for refinement. *R*-factors based on *F*^2^ are statistically about twice as large as those based on *F*, and *R*- factors based on ALL data will be even larger.

### Fractional atomic coordinates and isotropic or equivalent isotropic displacement parameters (Å^2^)

**Table:**

	*x*	*y*	*z*	*U*_iso_*/*U*_eq_	
S1	0.794704 (10)	0.52906 (6)	−0.01577 (5)	0.02346 (8)	
F1	0.79830 (2)	0.37874 (17)	0.48808 (16)	0.0338 (2)	
F2	0.94005 (2)	0.27091 (16)	0.53340 (11)	0.0261 (2)	
F3	0.92412 (3)	−0.07230 (15)	0.24104 (14)	0.0264 (2)	
F4	0.91204 (3)	0.60294 (17)	−0.07755 (14)	0.0338 (2)	
N1	0.78098 (4)	0.8994 (2)	0.14916 (18)	0.0236 (3)	
N2	0.81456 (4)	0.8012 (2)	0.22916 (17)	0.0209 (2)	
N3	0.85771 (4)	0.4805 (2)	0.21110 (16)	0.0190 (2)	
C1	0.76794 (4)	0.7789 (3)	0.0214 (2)	0.0249 (3)	
H1A	0.7458	0.8235	−0.0455	0.030*	
C2	0.82545 (4)	0.6085 (2)	0.15657 (18)	0.0160 (2)	
C3	0.87577 (4)	0.5273 (2)	0.3784 (2)	0.0187 (3)	
H3A	0.8637	0.6655	0.4249	0.022*	
H3B	0.9049	0.5537	0.3647	0.022*	
C4	0.86929 (4)	0.3330 (2)	0.50333 (18)	0.0162 (2)	
C5	0.83046 (4)	0.2629 (3)	0.5541 (2)	0.0213 (3)	
C6	0.82293 (5)	0.0864 (3)	0.6657 (2)	0.0242 (3)	
H6A	0.7963	0.0468	0.6955	0.029*	
C7	0.85629 (5)	−0.0312 (3)	0.7328 (2)	0.0238 (3)	
H7A	0.8520	−0.1523	0.8078	0.029*	
C8	0.89596 (5)	0.0308 (3)	0.68863 (19)	0.0220 (3)	
H8A	0.9184	−0.0466	0.7334	0.026*	
C9	0.90109 (4)	0.2105 (2)	0.57631 (19)	0.0181 (3)	
C10	0.87024 (4)	0.2743 (2)	0.1207 (2)	0.0194 (3)	
H10A	0.8557	0.2656	0.0126	0.023*	
H10B	0.8627	0.1417	0.1882	0.023*	
C11	0.91598 (4)	0.2685 (2)	0.08670 (19)	0.0175 (3)	
C12	0.94120 (4)	0.0961 (2)	0.14585 (18)	0.0184 (3)	
C13	0.98273 (5)	0.0831 (3)	0.1130 (2)	0.0234 (3)	
H13A	0.9984	−0.0374	0.1548	0.028*	
C14	1.00032 (4)	0.2553 (3)	0.0158 (2)	0.0260 (3)	
H14A	1.0282	0.2512	−0.0072	0.031*	
C15	0.97688 (5)	0.4331 (3)	−0.0475 (2)	0.0272 (3)	
H15A	0.9886	0.5489	−0.1125	0.033*	
C16	0.93555 (4)	0.4336 (2)	−0.0113 (2)	0.0218 (3)	

### Atomic displacement parameters (Å^2^)

**Table:**

	*U*^11^	*U*^22^	*U*^33^	*U*^12^	*U*^13^	*U*^23^
S1	0.01673 (15)	0.03179 (18)	0.02187 (15)	0.00300 (13)	−0.00547 (14)	−0.00224 (18)
F1	0.0139 (4)	0.0423 (5)	0.0451 (5)	0.0051 (4)	−0.0022 (4)	0.0137 (6)
F2	0.0124 (4)	0.0360 (5)	0.0298 (5)	0.0007 (3)	−0.0012 (3)	0.0101 (4)
F3	0.0237 (4)	0.0208 (4)	0.0345 (5)	0.0013 (3)	0.0037 (4)	0.0108 (4)
F4	0.0341 (5)	0.0282 (5)	0.0390 (5)	0.0040 (4)	−0.0034 (4)	0.0175 (4)
N1	0.0206 (6)	0.0248 (6)	0.0253 (6)	0.0062 (5)	−0.0010 (5)	0.0065 (5)
N2	0.0187 (6)	0.0213 (6)	0.0226 (6)	0.0044 (4)	−0.0020 (5)	0.0032 (5)
N3	0.0181 (5)	0.0192 (5)	0.0196 (5)	0.0039 (4)	−0.0049 (4)	−0.0013 (5)
C1	0.0165 (6)	0.0339 (8)	0.0243 (7)	0.0059 (6)	−0.0018 (5)	0.0044 (6)
C2	0.0127 (6)	0.0192 (6)	0.0161 (5)	−0.0015 (5)	−0.0012 (4)	0.0041 (5)
C3	0.0192 (6)	0.0172 (6)	0.0198 (6)	−0.0007 (5)	−0.0054 (5)	0.0006 (5)
C4	0.0132 (5)	0.0177 (6)	0.0177 (6)	0.0003 (4)	−0.0022 (5)	0.0000 (5)
C5	0.0150 (6)	0.0230 (7)	0.0258 (7)	0.0015 (5)	−0.0015 (5)	0.0003 (6)
C6	0.0199 (7)	0.0277 (8)	0.0250 (7)	−0.0054 (6)	0.0019 (5)	0.0003 (6)
C7	0.0296 (8)	0.0218 (7)	0.0200 (7)	−0.0022 (6)	0.0024 (6)	0.0032 (6)
C8	0.0220 (7)	0.0233 (7)	0.0206 (7)	0.0039 (6)	−0.0031 (5)	0.0030 (6)
C9	0.0141 (6)	0.0208 (6)	0.0193 (6)	−0.0005 (5)	−0.0004 (5)	0.0001 (5)
C10	0.0167 (6)	0.0180 (6)	0.0235 (7)	0.0011 (5)	−0.0027 (5)	−0.0023 (6)
C11	0.0167 (6)	0.0176 (6)	0.0181 (6)	0.0001 (5)	−0.0012 (5)	0.0001 (5)
C12	0.0203 (6)	0.0164 (6)	0.0185 (6)	−0.0009 (5)	0.0014 (5)	0.0005 (5)
C13	0.0201 (7)	0.0243 (7)	0.0259 (7)	0.0049 (5)	0.0009 (5)	−0.0005 (6)
C14	0.0184 (6)	0.0345 (8)	0.0252 (8)	−0.0026 (6)	0.0044 (5)	−0.0024 (7)
C15	0.0278 (8)	0.0299 (8)	0.0238 (8)	−0.0075 (6)	0.0042 (6)	0.0053 (6)
C16	0.0252 (7)	0.0191 (6)	0.0211 (6)	0.0009 (5)	−0.0027 (6)	0.0058 (7)

### Geometric parameters (Å, °)

**Table:**

S1—C1	1.7278 (16)	C6—C7	1.392 (2)
S1—C2	1.7431 (14)	C6—H6A	0.9300
F1—C5	1.3527 (16)	C7—C8	1.389 (2)
F2—C9	1.3630 (15)	C7—H7A	0.9300
F3—C12	1.3548 (17)	C8—C9	1.380 (2)
F4—C16	1.3566 (16)	C8—H8A	0.9300
N1—C1	1.295 (2)	C10—C11	1.5179 (19)
N1—N2	1.3872 (16)	C10—H10A	0.9700
N2—C2	1.3114 (19)	C10—H10B	0.9700
N3—C2	1.3613 (17)	C11—C12	1.3824 (19)
N3—C10	1.4570 (18)	C11—C16	1.388 (2)
N3—C3	1.4598 (19)	C12—C13	1.383 (2)
C1—H1A	0.9300	C13—C14	1.387 (2)
C3—C4	1.514 (2)	C13—H13A	0.9300
C3—H3A	0.9700	C14—C15	1.383 (2)
C3—H3B	0.9700	C14—H14A	0.9300
C4—C9	1.3848 (18)	C15—C16	1.380 (2)
C4—C5	1.3906 (19)	C15—H15A	0.9300
C5—C6	1.374 (2)		
			
C1—S1—C2	86.35 (7)	C9—C8—C7	118.05 (14)
C1—N1—N2	112.47 (13)	C9—C8—H8A	121.0
C2—N2—N1	112.07 (13)	C7—C8—H8A	121.0
C2—N3—C10	121.42 (12)	F2—C9—C8	117.87 (12)
C2—N3—C3	119.33 (12)	F2—C9—C4	117.72 (12)
C10—N3—C3	118.42 (11)	C8—C9—C4	124.41 (13)
N1—C1—S1	115.07 (11)	N3—C10—C11	112.32 (11)
N1—C1—H1A	122.5	N3—C10—H10A	109.1
S1—C1—H1A	122.5	C11—C10—H10A	109.1
N2—C2—N3	123.20 (13)	N3—C10—H10B	109.1
N2—C2—S1	114.03 (10)	C11—C10—H10B	109.1
N3—C2—S1	122.77 (11)	H10A—C10—H10B	107.9
N3—C3—C4	112.33 (11)	C12—C11—C16	114.68 (12)
N3—C3—H3A	109.1	C12—C11—C10	122.98 (12)
C4—C3—H3A	109.1	C16—C11—C10	122.31 (13)
N3—C3—H3B	109.1	F3—C12—C11	117.97 (12)
C4—C3—H3B	109.1	F3—C12—C13	117.77 (13)
H3A—C3—H3B	107.9	C11—C12—C13	124.25 (13)
C9—C4—C5	114.46 (13)	C12—C13—C14	117.94 (14)
C9—C4—C3	123.33 (11)	C12—C13—H13A	121.0
C5—C4—C3	122.21 (12)	C14—C13—H13A	121.0
F1—C5—C6	118.65 (13)	C15—C14—C13	120.85 (14)
F1—C5—C4	116.87 (13)	C15—C14—H14A	119.6
C6—C5—C4	124.48 (14)	C13—C14—H14A	119.6
C5—C6—C7	118.08 (14)	C16—C15—C14	118.03 (14)
C5—C6—H6A	121.0	C16—C15—H15A	121.0
C7—C6—H6A	121.0	C14—C15—H15A	121.0
C8—C7—C6	120.51 (14)	F4—C16—C15	118.49 (13)
C8—C7—H7A	119.7	F4—C16—C11	117.26 (12)
C6—C7—H7A	119.7	C15—C16—C11	124.24 (13)
			
C1—N1—N2—C2	0.11 (18)	C7—C8—C9—C4	−0.5 (2)
N2—N1—C1—S1	0.64 (17)	C5—C4—C9—F2	−179.53 (12)
C2—S1—C1—N1	−0.89 (12)	C3—C4—C9—F2	0.5 (2)
N1—N2—C2—N3	179.58 (13)	C5—C4—C9—C8	0.9 (2)
N1—N2—C2—S1	−0.80 (15)	C3—C4—C9—C8	−179.07 (14)
C10—N3—C2—N2	−176.38 (13)	C2—N3—C10—C11	131.34 (14)
C3—N3—C2—N2	14.2 (2)	C3—N3—C10—C11	−59.13 (17)
C10—N3—C2—S1	4.04 (19)	N3—C10—C11—C12	121.31 (15)
C3—N3—C2—S1	−165.40 (10)	N3—C10—C11—C16	−60.72 (19)
C1—S1—C2—N2	0.94 (11)	C16—C11—C12—F3	−179.37 (13)
C1—S1—C2—N3	−179.44 (13)	C10—C11—C12—F3	−1.3 (2)
C2—N3—C3—C4	113.44 (14)	C16—C11—C12—C13	0.1 (2)
C10—N3—C3—C4	−56.32 (16)	C10—C11—C12—C13	178.17 (14)
N3—C3—C4—C9	118.26 (15)	F3—C12—C13—C14	−179.93 (14)
N3—C3—C4—C5	−61.69 (18)	C11—C12—C13—C14	0.6 (2)
C9—C4—C5—F1	179.22 (13)	C12—C13—C14—C15	−0.6 (2)
C3—C4—C5—F1	−0.8 (2)	C13—C14—C15—C16	−0.1 (2)
C9—C4—C5—C6	−0.6 (2)	C14—C15—C16—F4	−177.79 (14)
C3—C4—C5—C6	179.38 (14)	C14—C15—C16—C11	0.9 (2)
F1—C5—C6—C7	−179.92 (14)	C12—C11—C16—F4	177.85 (13)
C4—C5—C6—C7	−0.1 (2)	C10—C11—C16—F4	−0.3 (2)
C5—C6—C7—C8	0.6 (2)	C12—C11—C16—C15	−0.9 (2)
C6—C7—C8—C9	−0.3 (2)	C10—C11—C16—C15	−178.99 (15)
C7—C8—C9—F2	179.95 (13)		

### Hydrogen-bond geometry (Å, °)

**Table:**

*D*—H···*A*	*D*—H	H···*A*	*D*···*A*	*D*—H···*A*
C1—H1A···N1^i^	0.93	2.58	3.392 (2)	146
C3—H3A···N2	0.97	2.36	2.8155 (18)	108
C3—H3B···F2	0.97	2.41	2.8508 (15)	107
C3—H3B···F3^ii^	0.97	2.47	3.0226 (16)	116
C8—H8A···F4^iii^	0.93	2.54	3.144 (2)	123
C10—H10A···S1	0.97	2.53	3.0738 (14)	115
C10—H10B···F3	0.97	2.40	2.8453 (16)	107
C10—H10B···N2^iv^	0.97	2.56	3.4191 (17)	148

Symmetry codes: (i) −*x*+3/2, *y*, *z*−1/2; (ii) *x*, *y*+1, *z*; (iii) *x*, *y*−1, *z*+1; (iv) *x*, *y*−1, *z*.
